# Neuromuscular blocking agents for acute respiratory distress syndrome: how did we get conflicting results?

**DOI:** 10.1186/s13054-019-2586-3

**Published:** 2019-09-06

**Authors:** Heather Torbic, Sudhir Krishnan, Abhijit Duggal

**Affiliations:** 1 Department of Pharmacy, Cleveland Clinic, 9500 Euclid Avenue, Hb-105, Cleveland, OH 44195 USA; 2 Department of Critical Care, Respiratory Institute, Cleveland Clinic, 9500 Euclid Avenue, L2-330, Cleveland, OH 44195 USA

Letter

Except for neuromuscular blocking agents (NMBAs), pharmacologic therapies studied for ARDS have not demonstrated mortality benefit. By improving ventilator synchrony, NMBAs reduce ventilator-induced lung injury (VILI) and aid in lung recruitment, thereby improving oxygenation. NMBAs exert their benefit in ARDS by decreasing systemic inflammation and improving V/Q mismatch [1]. The seminal trial, ACURASYS, evaluated the use of NMBAs for early, moderate-severe ARDS. It reported an adjusted 90-day mortality benefit of 9.1% with a reduction in the incidence of VILI in patients with a PaO_2_/FiO_2_ < 120 [2]. Despite these findings, enthusiasm for NMBA use in ARDS remained lackluster [3].

To dispel the concerns associated with adoption of NMBAs, the recently published ROSE trial evaluated the use of NMBAs for early, moderate-severe ARDS. It reported no difference in 90-day mortality or incidence of VILI [4]. The conflicting results of the two major trials bring to question the role of NMBAs in the management of ARDS.

The divergent results could be accounted for by various factors (Table 1). First, ARDS was defined differently in both trials. Although patients were enrolled with PaO_2_/FiO_2_ < 150 mmHg, ROSE patients had a higher baseline PEEP (≥ 8 cm H_2_O). Second, patients were enrolled 8 h earlier in ROSE [8(4–16) vs. 16(6–29) h] [2, 4]. Patients who would have been excluded from ACURASYS (those who rapidly improved before randomization) were likely included in ROSE limiting the trial’s effect. Third, both non-pharmacologic and pharmacologic management was different. Patients in both arms of ACURASYS were deeply sedated, whereas only patients in the treatment arm of ROSE were deeply sedated. Deep sedation, especially in early ARDS, is associated with reverse triggering, which can result in breath stacking, VILI, and increased mortality. Reverse triggering and increased risk of ICU delirium, with decreased time to extubation secondary to deep sedation, may have resulted in higher mortality in the control arm in ACURASYS compared to those in the control arm of ROSE with lighter sedation targets [5]. Finally, both studies protocolized ventilator management and lung protective strategies. However, patients in ROSE received lower FiO_2_, but higher PEEP and lower tidal volumes in both study arms, probably improving lung recruitment and decreasing atelectrauma [5].

**Table 1:** Differences between ACURASYS and ROSE trials

	ACURASYS (2)	ROSE (4)
ARDS definition	-AECC	-Berlin
Median time to enrollment from ARDS diagnosis	-16 (6–29) vs. 18 (6–31) h	-8.2 (4.0–16.4) vs. 6.8 (3.3–14.5) h
Sedation targets	-Control arm—goal Ramsay score 6 (deep sedation)	-Control arm—goal Ramsay score 2–3 (light sedation)
Ventilator strategies	-TV 6–8 ml/kg -Low PEEP (≥ 5 cm H_2_O)	-TV 6 ml/kg -High PEEP (≥ 8 cm H_2_O)
Crude 90-day mortality	-31.6% vs. 40.7%, *p* = 0.08	-42.5% vs. 42.8%; *p* = 0.93
28-day mortality	-23.7% vs. 33.3%, *p* = 0.05	-36.7% vs. 37.0%, *p* = NS
Ventilator induced lung injury	-Barotrauma: 5.1% vs. 11.7%; *p* = 0.03 -Pneumothorax: 4.0% vs. 11.7%; *p* = 0.01	-Barotrauma: 4.0% vs. 6.3%; *p* = 0.12 -Pneumothorax: 2.8% vs. 5.0%; *p* = 0.10
Other adverse effects	-None	-Serious cardiovascular events: NMBA14 vs. Control 4; *p* = 0.02

*AECC* American-European Consensus Conference, *ARDS* acute respiratory distress syndrome, *TV* tidal volume, *PEEP* positive end-expiratory pressure, *NMBA* neuromuscular blocking agent

The differences in study design and methodology best explain the varied results and any inference drawn; comparing these two trials would be misleading. In light of these recent findings, interest in the use of NMBAs might be tempered. NMBAs may have a role in ARDS management in the correct clinical context (refractory hypoxemia and recalcitrant ventilator dyssynchrony). Non-pharmacologic strategies centered around lung-protective ventilation and PEEP optimization should continue to be the backbone of ARDS management.

## Data Availability

1. Papazian L, Forel JM, Gacouin A, Penot-Ragon C, Perrin G, Loundou A, et al. Neuromuscular blockers in early acute respiratory distress syndrome. *N Engl J Med*. 2010; 363(12):1107–1116. 2. Moss M, Huang DT, Brower RG, Ferguson ND, Ginde AA, Gong MN, et al. Early neuromuscular blockade in the acute respiratory distress syndrome. *N Engl J Med*. 2019; 380(21):1997–2008.
